# Cost-Effectiveness of Tele-Video-Consultation for the Neuro-Surgical Emergency Management at the General Hospitals in Italy

**DOI:** 10.3389/fnins.2018.00908

**Published:** 2018-12-04

**Authors:** Rajendra Kadel, Sara Evans-Lacko, Andrea Tramarin, Giampaolo Stopazzolo

**Affiliations:** ^1^Personal Social Services Research Unit, London School of Economics and Political Science, London, United Kingdom; ^2^Institute of Psychology, Psychiatry and Neuroscience, King's College London, London, United Kingdom; ^3^ULSS 6, Azienda Sanitaria di Vicenza, Vicenza, Italy

**Keywords:** cost-effectiveness analysis, tele-video-consultation, neuro-surgery, emergency case management, Italy

## Abstract

**Background:** Neuro-surgical emergencies are serious (long-term disability and high mortality) and costly to the national health services. Tele-medicine intervention can facilitate to reduce this gap. Our study aims to evaluate the cost-effectiveness of tele-video-consultation intervention for the management of neuro-surgical emergencies in the general hospitals.

**Methods:** We retrieved health service data from the tele-consultation service, online tele-medicine database portal and hospital patient registry, between January 2009 and December 2012 and evaluated cost-effectiveness of the tele-video-consultation intervention from an Italian National Health Service perspective.

**Results:** Seventy-five percent of the tele-consultations were completed within 15 min and 90% within 30 min. The average costs were €2,326 in the intervention group and €4,173 in the care as usual group. The intervention avoided 73% potential transfer (saving of 139,916 km travel distance during a 4-years period). The incremental cost-saving per transfer avoided from the tele-medicine intervention was €365.

**Conclusions:** Tele-medicine intervention could be worth investing from the Italian National Health Service perspective.

## Introduction

Neurosurgical emergencies are costly to the health care systems as well as to the patient, and these are serious in terms of mortality and long-term disability. Tele-medicine intervention can be effective and worth investing for the management of such emergencies. However, such evidence has not been well-established. This study aims to evaluate the cost-effectiveness of tele-medicine intervention for the management of neuro-surgical emergencies from the Italian health care perspective.

The literature showed that traumatic brain injury (TBI), vertebral fracture, spinal cord injury (SCI), intracranial hemorrhage (ICH) and peripheral nerve injury are the common form of neuro-surgical emergencies, among them, TBI is a frequent cause. It has been estimated that about 2.5 million Europeans experience some forms of TBI, among them one million would be admitted to the hospitals and 75,000 would die (Maas et al., 2015). The TBI rates were higher in males than its female counterparts, and the rates of hospitalization and mortality were higher among elder patients (Faul et al., 2010). In the USA, 1.5 million people were experiencing TBI and 50,000 of them would die every year (National Institute for Health, 2013). In the Netherland, an estimated 7.1 disability adjusted life-years (DALYs) lost per case was associated with TBI (Scholten et al., 2014). In Europe, the incidence of vertebral fracture was 12.1 per 1,000 person-year in female and 6.8 per 1,000 person-year in males (Felsenberg et al., 2002), SCI ranged from 10.7 to 120 per million population (Furlan et al., 2013) and ~300,000 peripheral nerve injuries occurred every year (Hart, 2003). In the Western countries, the cases of ICH ranged from 10 to 30 per 100,000 population (Flaherty et al., 2010). The incidence of cancer of the brain and central nervous system were estimated at 7.8 per 100,000 population with the mortality of 6 cases per 100,000 population in the European countries in 2012 (World Health Organization, 2012).

The costs for the management of neuro-surgical emergencies are substantial, but the evidence is limited. For example, an estimated costs of TBI management was €33 billion (2010 €) per year in Europe (Olesen et al., 2012) and $56 billion per year in the USA (National Institute for Health, 2013). The average inpatient costs of TBI ranged from €2,529 in Germany, €2,833 in Spain to €3,024 in Sweden, and an additional cost of ~€1,000 for a patient with a concussion, and additional €6,000 for a patient with severe brain injury (Berg et al., 2005). The lifetime TBI mortality costs were estimated at €375,000 per death in Sweden (Berg et al., 2005). In the Netherlands, the total costs of TBI were estimated at €314.6 million per year (Scholten et al., 2014). The total costs of stroke in Europe were estimated at €37,412 million (2010 €) per year (Olesen et al., 2012). Similarly, a study from a developing country showed that the average costs of managing traumatic brain injury secondary to road traffic accident was $7,365 (You et al., 2018).

The incidence of neuro-surgical emergencies is high, and complications are serious which incur substantial costs. The risk of mortality and complications are increasing over time, and hence early and planned management of such emergencies is crucial (Tasker et al., 2006). Studies also revealed that the majority of neuro-surgical cases do not require surgical treatment (Esposito et al., 2005), and can be managed in the general hospitals by trained medical doctors in coordination with the distant specialists. Telemedicine can be supportive to reduce this gap. Telemedicine can help to reduce unnecessary transfer and can improve effective management of the severe cases at referral hospitals. It can also help to reduce secondary insults resulting from unnecessary transfer. Few referral cases mean less patient transfer and less treatment reimbursement costs. This can save substantial economic costs to the general hospitals. Moreover, a planned referral can also help to improve service quality and treatment outcomes.

We conducted a quasi-experimental study to evaluate the cost-effectiveness of tele-consultation intervention for the management of neuro-surgical emergencies. The intervention, based on teleconsultation project developed and implemented in the north-eastern part of Italy, was added to care as usual (CAU) and compared it with CAU only.

## Material and Methods

### Study Setting

The study was carried out from the hospitals of the two local health authorities (two hospitals in one health authority and four hospitals in second health authority and each health authority has a capacity of about 600 hospital beds) and one specialist referral hospital in the north-eastern part of Italy. We retrieved real-time tele-consultation service data and hospital patient registry data between 1st January 2009 and 31st December 2012.

### Participants

Participants were men and women from all age groups, who attended neurological unit of the respective hospitals from two local health authorities, irrespective of gender, race, geography and socioeconomic groups. Patients who needed neuro-surgical tele-consultation were classified into traumatic and non-traumatic category through medical triage. Patients were followed up until discharge from the hospitals after tele-consultation, or death. Overall, 1,676 patients were consulted with specialists for tele-neurosurgery from the two local health authorities between 2009 and 2012.

### Intervention

A multi-specialist tele-video-consultation system with the dedicated network was installed on the workstations (hospitals) of the two local health authorities (spoke hospitals) and one specialist hospital (hub hospital) in the Veneto region of Italy. The software for tele-consultation service enables the operator to perform data compilation, digital signatures, video-conference and sharing of diagnostic images. The application enables providers with a real time video consultation and digitalized radiological image transfer facility. The software automatically updates all the information once these are recorded into the online database portal. The consultant neuro-surgeon reviews the information after the patient information along with radiological images are uploaded to the systems, then real-time video-consultation or in some cases, offline communication (by telephone) are established to the referring doctor (usually, a neurologist) for medical and transfer decisions. Based on the recommendation, a local doctor make a transfer decision. Those who are not transferred have been managed at the local hospitals based on local clinical guidelines.

The main purpose of this intervention was to reduce potential patient transfer, well-managed hospital admission and improvement of patient health outcomes. The tele-medicine intervention added-up with CAU was compared to the CAU only for the management of neuro-surgical emergencies. The CAU was the hypothetical control group without tele-medicine services. In the CAU group, all potential cases would be transferred to the referral hospital for the specialist treatment as potential tele-consultation cases were deemed to be transferred in the absence of tele-neurosurgery intervention. Both the intervention and CAU groups received standard medical care based on local clinical guidelines.

### Outcome Measures

The primary outcome measures were tele-consultation response time and potential transfer avoided. The response time was measured in minutes and this was operationalized as the time between the successful update of patient information and radiological images on the database portal by the referring physician, and the response to the tele-consultation by the consultant neurosurgeon with the recommended transfer decision. The date and time of every tele-consultation events were updated automatically in the online database portal. Potential transfers avoided was measured in percentage terms and travel distance in kilometers.

### Service Use and Costs

The cost of intervention was estimated from an Italian national health service perspective. The service use costs were discounted at the annual rate of 3% as recommended by the Italian economic evaluation guidelines (Capri et al., 2001), but we have not discounted the health benefits as some authors argued that health benefits should not be discounted (Hillman and Kim, 1995). The price index year was 2009 in Euros.

We assessed the following costs: direct health service costs, tele-medicine technology and maintenance costs, patient transfer and attending staff costs, additional costs of treating non-transfer cases at the general hospitals and consultation fees, but facility charges and human resource costs of the local hospitals were not included for cost estimation as the new intervention utilized existing facilities and human resources. The medical and service use costs of referral patients were reimbursed to the referral hospital according to Italian diagnosis related group (DRG) cost reimbursement guidelines. The service installation costs and maintenance charges were collected from the tele-medicine coordination office. The costs of internet service use were estimated based on monthly subscription fees. The tele-consultation fees were calculated based on unit cost per consultation. The transfer costs were estimated based on unit cost per kilometer multiplied by the total distance traveled. For referral service, attending staff included one medical doctor, one nurse and one driver. The costs of attending staff were estimated based on unit cost per hour multiplied by total time needed to transfer patients to the specialist hospital. The additional costs of treatment of non-referral cases were estimated based on average medicines and related costs for an average period of 7 days per patient.

### Statistical Analysis

Patient related data were retrieved from the tele-medicine database portal and the hospital patient registry over a 4-years period. The general study characteristics were analyzed using descriptive statistics. Linear regression method was used to perform subgroup analysis to address parameter uncertainty. We used chi-square test to examine the variability in mortality by age group. The intervention data (effects and costs) were analyzed using Stata version 12 (www.stata.com).

We assessed the variability in tele-consultation response time by types of neuro-surgical case and local health authority using *t*-test. We also analyzed neuro-surgical mortality by risk factors (e.g., age, gender, and length of hospital stays) using z-test to see whether there was subgroup variation in the risk of mortality among neuro-surgical cases. Similarly, we regressed mean DRG costs by patient's age, gender, length of hospital stays and disability rating at discharge to examine the relationship of DRG mean costs with these variables.

The incremental cost-effectiveness ratios were calculated in terms of cost per transfer avoided. The unit cost of the intervention was calculated based on item-wise cost data from resource use data and patient registry.

### Cost-Effectiveness Analysis

The main aim of this study was to examine whether tele-consultation intervention was cost-effective and cost saving when added to CAU for the management of neuro-surgical emergencies. The cost-effectiveness analysis was conducted from an Italian National Health Service perspective. Health and social care costs (e.g., patient transfer costs) for the period of 4 years starting from January 2009 were examined alongside the tele-consultation database portal and hospital registry data from both spokes and hub hospitals. We evaluated cost-effectiveness of tele-consultation intervention based on single outcome measures (cost per potential transfer avoided).

## Results

### General Characteristics

A total of 1,676 tele-consultation were made for emergency neuro-surgical cases between general hospitals of two local health authorities and specialist hospital from 1st of January 2009 to 31st of December 2012. Out of the total cases, around 79% were traumatic neuro-surgical cases. The data showed that there was an increment in the cases of neurosurgical teleconsultations at 17.12% in 2009 and 31.62% in 2012.

In 75% of the cases, tele-consultation was completed (response time) within 15 min and 90% were completed within 30 min. Response time did not differ by type of neuro-surgical cases (*t*: −0.52, *p* = 0.603) or by local health authority (*t*: 0.12, *p* = 0.905). Use of tele-medicine intervention was associated with 73% decrease in transfer rate to the specialist hospital. This was equivalent to 139,916 km during a 4-years period.

Among referral patients (*n* = 455), the youngest patient was 4 years old and the oldest patient was 94 years old, with a mean age of 63 years (SD: 19.77). The mean observation time was 23 h, which was the time between patient's name entry and admission to inpatient ward of a hospital. The length of hospital stay was an average of 8 days (SD: 6.5 days) that ranged from hours to months. The average cost of treatment was €6,965 (SD: €6,002.92) with a minimum of €200 and the maximum of €37,123.

Thirty-six referral cases (2.5%) died during treatment period, 34.95% cases discharged home with complete treatment, around 54% transferred to other hospitals either to continue existing treatment or further treatment, and the remainders were discharged at home with nursing care. Death of patients was associated with greater age (death at age group 80 and above; *p* = 0.036), and length of hospital stay less number of deaths with longer hospital days (hospital stay 1–2 weeks; OR: 0.26, *p* = 0.010). Female had a lower chances of mortality risk, but the difference was not statistically significant (OR: 0.64, *p* = 0.265).

**Figure 1:**
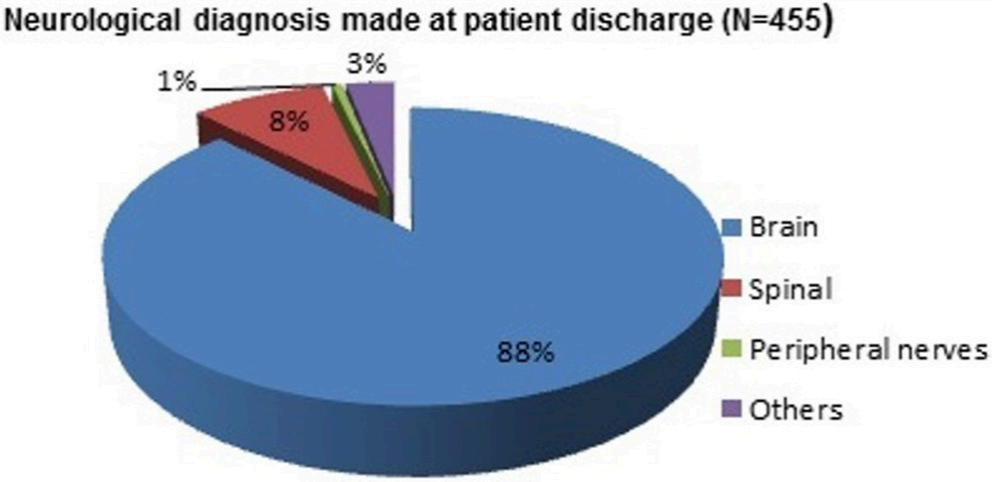
Neurosurgical diagnosis at discharge.

Neuro-surgical diagnoses are presented in Figure 1. Most of the neuro-surgical diagnoses were brain related disorders (88%, *n* = 400), such as brain cancer, brain injury or brain trauma. Eight percent (*n* = 36) cases were related to spinal problems, 3% (*n* = 14) with peripheral nerve problems and the remaining 1% (*n* = 5) were other neurological cases.

### Resource Use and Costs

Based on the limited information available, we estimated resource use and costs of intervention for the management of emergency neuro-surgical cases, which are summarized in Table 1. The major cost items were technology installation and maintenance, internet service, tele-consultation fees, patient transfer, DRG reimbursement and management of non-referral cases in the local hospitals. An estimated average cost of neurosurgical service was €2,326 per patient in the intervention group and €4,173 per patient in CAU group.

**Table 1:** Use of health care resources and costs for emergency neuro-surgical case management in Tele-medicine intervention.

**Parameters**	**Unit cost €**	**Unit of measure**	**Source**	**CAU**		**Tele-consultation**	
				**Unit**	**Mean cost €**	**Unit**	**Mean cost €**
Technology installation	8,125	Sets	A	–	–	8	65,000
Technology maintenance	30,000	Yearly	A	–	–	4	120,000
Internet service	4,800	Yearly	A	–	–	4	19,200
Tele-consultation	120	Episode	A	–	–	1,676	201,120
Total	242				0	1,676	405,320
**TRANSFER/TRAVEL**							
Ambulance	2	Km	B	190,344	380,688	50,428	100,856
Doctor	100	Hourly	B	4,237	423,700	1,128	112,800
Nurse	50	Hourly	B	4,237	211,850	1,128	56,400
Driver	40	Hourly	B	4,237	169,480	1,128	45,120
Total							315,176
Total with technology					1,185,718		720,496
Discounted					1,134,913		689,625
**DRG (REFERRAL CASES)**							
Surgical	8,246	Case	C	355	2,927,330	355	2,927,330
Nonsurgical	2,418	Case	C	1,321	3,194,178	100	241,800
Total				1,676	6,121,508	455	3,169,130
Management of non-transfer cases	150	Case	B	–	–	1,221	183,150
Grand total				1,676	7,307,226	1,676	4,072,776
Discounted					6,994,132		3,898,269
Cost per patient (discounted)					€4,173		€2,326

*Sources of information used in the cost analysis, (a) Tele-medicine coordination office, (b) local hospital and Tele-medicine coordination office source, (c) referral hospital patient registry. Base year = 2009*.

The descriptive analysis showed that the DRG cost of surgical cases was much higher (Mean: €8,246, SD: €6,178) than non-surgical cases (Mean: €2,418, SD: €1,415). Similarly, there was a difference in the length of hospital stay between surgical (Mean: 8 days, SD: 7 days) and non-surgical (Mean: 6 days, SD: 4 days) cases. Details of DRG costs and length of hospital stay due to neuro-surgical causes are summarized in Table 2.

**Table 2:** Average DRG cost and hospital stay days by neuro-surgical group.

**Variable**	**Obs**.	**Mean**	**Std. Dev**.	**Min**	**Max**
Cost of surgical case	355	8,245.61	6,178.12	200	37,123.09
Cost of non-surgical case	100	2,418.01	1, 415.36	214.93	7, 603.26
Days of hospital stay (surgical case)	355	8.38	7.05	< 1	73.00
Days of hospital stay (non-surgical case)	100	6.20	3.90	1.00	17.00

The study showed that age and gender were not predictive of DRG costs for neuro-surgical treatment. There was a statistically significant difference in the DRG costs and length of hospital stay- longer stay more cost (hospital stay more than 7 days Mean DRG cost: €2,871.39, 95% CI: 1,762.36; 3,980.43, *p* < 001). Similarly, DRG cost was associated with severity of disability level at discharge—the higher costs with an increased level of disability (for example, people with no disability at discharge cost €4,132.82 less per person as compared to patients with total dependence at discharge).

### Cost-Effectiveness Analysis

Table 3 shows the cost-effectiveness analysis of tele-medicine intervention. Our study findings suggested that tele-medicine intervention for emergency neuro-surgical case management was cost saving and cost-effective as compared to CAU. We estimated a total cost of €3,898,269 (2009 €) in the intervention group and €6,994,132 (2009 €) in the CAU group for the management of 1,676 neuro-surgical patients at each group. The intervention saved a total of €3,095,863 which was equal to an average saving of €1,842 per person compared to CAU group.

**Table 3:** Cost-effectiveness analysis results.

**Effects**	**Mean difference, ICER, ACER**
Potential transfers avoided as an outcome (*n* = 1,676)
Incremental costs (€)^*^	– €445,288
Incremental transfers avoided	1,221
ICER (€per transfer avoided)	– €365 per transfer avoided (Intervention dominated)

ICER, incremental cost-effectiveness ratio; net costs, total costs–intervention costs. Costs were estimated at 2,009 prices.

* *In the intervention arm, technology installation and maintenance costs, internet service costs, and tele-consultation costs were also included in addition to transfer costs*.

We found that 73% (1,221 cases) neuro-surgical cases did not require referral for specialist treatment using tele-consultation service. A net savings from reducing potential transfer was €445,288 to the local health authorities over a 4-years period. The intervention avoided average costs of €365 per patient by reducing potential transfer.

## Discussion

We evaluated cost-effectiveness of the tele-neurosurgery intervention, delivered over a period of 4 years, added with care as usual and compared it with care as usual only. We found that the tele-medicine intervention was cost-effective and cost saving from an Italian National Health Service perspective. The intervention was dominant to avoid potential transfer costs associated with neurosurgical emergencies.

As with any other interventions, our study also has several limitations. We rely on the hospital patient registry and online tele-medicine database portal to collect data for analysis. We made some assumptions related to costs information in the control group. We did not perform sensitivity analysis to examine the robustness of the cost-effectiveness results. The control situation was a without scenario (the absence of tele-medicine service). As the study did not perform the random allocation of intervention and control participants, the study might be confounded by selection bias. We included all participants in the intervention group who visited hospital due to neuro-surgical emergencies and consulted through tele-medicine service during a 4-years period which was the strength of the intervention.

There is limited evidence on the cost-effectiveness of tele-medicine interventions for the management of neuro-surgical emergencies. Available studies showed a considerable reduction in potential transfer by the use of tele-medicine in neurosurgery: 37% potential transfer avoided in the study by Hassan et al. (2014), 44% in the study by Moya et al. (2010), 50% in the study by Heautot et al. (1999), 64% in the study by Chodroff (1999) and 67% in the study by Kreutzer et al. (2008). Similarly, some studies reported costs information: tele-radiology avoided 60% potential transfer costs in a study by Loose (2000), tele-medicine intervention reached breakeven point in a 15 months period with 282 neuro-surgical management (Kreutzer et al., 2008), a community tele-medicine intervention resulted in a net saving of $561,774 during 35 months period (Chodroff, 1999). A study from developing country also showed considerable savings with the help of tele-neurosurgery by reducing avoidable transfer (Hassan et al., 2014). These studies support our study findings. Above mentioned studies also showed favorable intervention outcomes for the improvement in clinical symptoms, reduction in disease severity and reduction in secondary events facilitated by tele-medicine service. It was also noticed that there was no difference in treatment outcomes to manage mild neurosurgical cases in the non-neurosurgical facility with the help of tele-neurosurgery compared to specialist hospital (Sidek et al., 2018).

The study limitations need to be considered when assessing its generalizability. First, we estimated cost-effectiveness of the intervention from an Italian National Health Service perspective, not from a societal perspective, which excluded indirect costs and out of pocket costs incurred by patients or family. Secondly, the study lacks baseline information about study participants, and we did not perform sensitivity analysis to produce robust results on cost-effectiveness.

Our study findings suggest that tele-medicine intervention for the management of neuro-surgical emergencies were cost-effective and cost-saving. The cost-effectiveness arises mainly due to avoiding the potential transfer. It seems that the intervention facilitates the effective management of neuro-surgical emergencies. And, it could be the case that tele-medicine intervention facilitates the reduction in secondary (further) complications and improved better management of neurosurgical cases through a reduction in unnecessary transfer and improve planned referral of neuro-surgical cases.

Emergencies are inevitable. Neuro-surgical emergencies are serious in terms of morbidity, mortality and costs (expensive in terms of transfer and treatment costs). Neuro-surgical events can happen to any people irrespective of age, gender and socioeconomic status. Early and effective management can reduce further complications, disease severity and mortality as well as reduction in substantial costs. Information technology in health care management can facilitate the effective management of neuro-surgical cases in the general hospitals in coordination with medical/surgical specialist at a distant. In these circumstances, tele-medicine intervention could be an effective approach to reduce morbidity and mortality associated with neurosurgical emergencies, and which considerably reduce overall health care costs should be made available for its wider applications.

## Ethics Statement

This paper is a part of a master thesis work. The main author was granted access available data for the study from the hospital authority. Fully anonymised patient data were retrieved from the patient registry of the specialist hospital in Italy. The data management process was fully compliance with the European data protection directives 1995.

## Author Contributions

RK drafted the manuscript. SE-L, AT, and GS subsequently revised this manuscript. All authors agreed on the final manuscript.

### Conflict of Interest Statement

SE-L received consulting fees from Lundbeck. The remaining authors declare that the research was conducted in the absence of any commercial or financial relationships that could be construed as a potential conflict of interest.
